# Temporal attentional bias to food stimuli in adolescent anorexia nervosa – an ERP study

**DOI:** 10.1186/s40337-026-01800-w

**Published:** 2026-10-22

**Authors:** Julia Nannt, Ines Wolz, Jan Vagedes, Jennifer Svaldi

**Affiliations:** 1 Clinical Psychology and Psychotherapy, University of Tübingen, Schleichstr. 4, 72076 Tübingen, Germany; 2 ARCIM Institute, Im Haberschlai 7, 70794 Filderstadt-Bonlanden, Germany; 3 Department of Neonatology, University Hospital, University of Tübingen, Calwerstr. 7, 72076 Tübingen, Germany; 4 German Center for Mental Health (DZPG), Schleichstr. 4, 72076 Tübingen, Germany

**Keywords:** Anorexia nervosa, Attentional bias, Event-related potential, Adolescence

## Abstract

**Background:**

Anorexia nervosa (AN) is associated with substantial psychological and physical impairment and low recovery rates. A deeper understanding of its underlying mechanisms is therefore crucial. Cognitive models of eating disorders emphasize the role of attentional biases in the processing of food stimuli. However, only a few studies to date have examined the temporal dynamics of food-related attentional bias in adolescents with AN.

**Methods:**

The present study combined the high temporal resolution of event-related potentials with the behavioral measure of the attentional blink in a rapid serial visual presentation task in order to disentangle the temporal processing stages that underlie food-related attentional biases in female adolescents with AN (*n* = 33) compared to age-matched healthy controls (*n* = 36).

**Results:**

Behavioral results revealed an increased attentional *engagement* with food in AN, but no impairment in attentional *disengagement*. Additionally, high-calorie food distractors elicited reduced early electrocortical processing followed by enhanced later processing in AN. These AN-specific attentional biases were no longer evident for target processing.

**Conclusions:**

The findings suggest that attention allocation to food in AN is a temporally dynamic process rather than a unitary mechanism. They further suggest that these biases are not entirely autonomous but rather depend on the availability of attentional resources.

**Supplementary information:**

The online version contains supplementary material available at https://doi.org/10.1186/s40337-026-01800-w.

## Abstract (plain language summary)

Anorexia nervosa (AN) typically emerges during adolescence and can have serious physical and psychological consequences. Because recovery is often difficult, researchers try to better understand the processes that maintain the disorder. This study looked at how female adolescents with AN compared with adolescents without an eating disorder attend to food images. We found that patients with AN paid more attention to food than the control group. Additionally, when we looked at the brain activity, the two groups differed: in females with AN, the initial brain response to high-calorie food images was weaker, but later it was stronger. This suggests that attention to food in AN is not a fixed pattern but unfolds and changes over the course of a few seconds. Interestingly, when the task was made more difficult and required more concentration, adolescents with AN showed no longer this biased attention to food. These results suggest that the way adolescents with AN attend to food depends on the mental capacity they have available at the moment. Future treatment approaches should take these factors into account.

**Supplementary information:**

The online version contains supplementary material available at https://doi.org/10.1186/s40337-026-01800-w.

## Background

The main symptoms of anorexia nervosa (AN) are restriction of food intake resulting in a significantly low body weight, an intense fear of an increase in body weight and anxiety or distress related to one’s body weight or shape [1]. AN is accompanied by severe functional impairment and has a profound impact on both psychological and physical health [2], with a tendency to persist over time, even after treatment [2–4]. In up to 36% of cases, the illness takes a chronic course [2]. Approximately 50% of adolescents who have undergone treatment for AN continue to display symptoms both immediately and one year following treatment termination [5, 6]. Given the significant impairments and low rates of recovery associated with adolescent AN [7], it is crucial to better understand its underlying mechanisms to improve current gold standard treatment.

In accordance with cognitive behavioral theories, pathological eating behavior results from negative schemas regarding food, the body and the self [8, 9]. Once activated, the theory predicts that disorder-relevant stimuli (e.g., food) from the environment are processed in a schema-consistent manner, for example through an altered attention allocation towards these stimuli [9, 10]. Notably, depending on the time course in the information processing stream, these attentional biases may comprise both an increased attention allocation towards and an increased avoidance of these stimuli, thereby resulting in behavioral symptoms including restrictive eating and compensatory behavior such as purging and excessive exercising [9].

Several studies have investigated attentional biases in AN. For the most part, these studies have employed paradigms that focused on the spatial distribution of food-related attention by means of reaction time and eye-tracking measures [11]. In adults with AN, generally, the results indicate an *early orientation* towards followed by a *subsequent avoidance* of food during processing of picture pairs of food and neutral stimuli [12–16]. By contrast, adolescents with AN showed an *early avoidance* of food [17] along with an *increased* attentional processing of food in later time windows indicating enhanced top-down processing [15]. Taken together, paradigms that assess the *spatial* allocation of attention during food processing yield indirect evidence of distinct attentional profiles throughout the information processing stream in (adolescent and adult) AN.

In addition to the spatial distribution of attention, the *temporal* processing of food – that is, how continued attention to food-related information influences the processing of subsequent information – provides another significant dimension to this approach. Previous research has demonstrated, that after attending to and during processing of a stimulus there is an impairment of perceptual abilities for detecting a subsequent stimulus, a phenomenon known as the attentional blink [18–20]. This attentional blink (i.e., the temporary lapse in perception during which one stimulus is missed) is thought to result from attention selection due to the brain’s natural capacity limitation [18, 20]. Notably, previous fundamental research has shown that salient stimuli capture attention in a stronger manner than neutral stimuli, thus modulating both magnitude and time course of the attentional blink [18].

Typically, the attentional blink phenomenon has been studied by the rapid serial visual presentation (RSVP) paradigm [18]. In the RSVP, a stream of filler stimuli contains two marked stimuli: a distractor and a target. Once the distractor is correctly identified, the detection of the target is diminished for up to the first 500 ms after distractor onset [20]. Emotionally salient stimuli modulate (i.e., reduce or enhance) the attentional blink [18]. Specifically, studies showed that if the *target* is emotionally salient to the participant (e.g., a spider in a spider phobic [21], or a specific substance in an individual with a substance use disorders [22–24]), the likelihood of detecting this target shortly after a neutral distractor (e.g., a pencil) increases compared to the detection of a neutral target (e.g., a stapler). This effect is known as *increased engagement*. Conversely, studies showed that if the *distractor* is salient for a participant (e.g., negative information in an individual with dysphoria [25] or food in an hungry individual [26, 27]) and therefore elicits intensified processing, a subsequently presented neutral target (e.g., a pencil) is detected less frequently than targets following neutral distractors (e.g., a stapler). This effect is known as *impaired disengagement*. Thus, increased engagement is evidenced by a reduced attentional blink, and impaired disengagement by an increased attentional blink. At the behavioral level, increased engagement results in an enhanced target accuracy, while impaired disengagement results in a reduced target accuracy.

To our knowledge, only one study to date has examined attentional processing in AN using the RSVP paradigm with food and neutral distractors and neutral targets [28]. Individuals with AN showed a significantly stronger attentional blink following food distractors compared to healthy controls (HC), suggesting a prolonged processing of disorder-relevant stimuli. Note however that this study focused exclusively on attentional disengagement. Therefore, it remains unclear whether AN is also characterized by altered attentional engagement. Furthermore, the study by Neimeijer et al. [28] relied exclusively on behavioral outcomes as a rather indirect measure of attention allocation [29] that cannot disentangle the different stages at which these effects emerge [30]. Specifically, behavioral data alone does not reveal whether group differences arise during early bottom-up processing, i.e., the allocation of attentional resources, or later top-down motivational processes.

To investigate the temporal dynamics of attentional processing, event-related potentials (ERPs) measured in electroencephalography (EEG) provide crucial markers for different stages of attention due to their excellent temporal resolution [31]. Additionally, ERPs can be effectively used to analyze targets presented serially and rapidly, thereby facilitating the discrimination of their respective processing dynamics [32]. Thus, ERPs are well-suited to differentiate rapid, automatic from later, resource-dependent attentional processes.

The early posterior negativity (EPN) is typically interpreted as an index of early, automatic attentional engagement [33]. Previous research has consistently shown that the EPN is a robust marker of enhanced perceptual processing for motivationally relevant stimuli [34, 35]. Two previous studies using passive viewing paradigms examined the EPN to food in individuals with AN. In one of these studies [36], food presented in a rapid picture stream elicited heightened EPN amplitudes compared to neutral stimuli in AN, suggesting an automatic attentional bias toward food. This increased automatic processing was mostly pronounced in high-calorie food. However, in the other study [37] female adolescents with AN showed an early avoidance of high-calorie food when presented for a prolonged duration as indicated by a reduced EPN.

In contrast to the EPN, the P300 is an ERP that is associated with later, resource-dependent stages, including the allocation of attention to stimuli that reach conscious awareness [31]. Within the attentional blink paradigm, the competition for limited resources is reflected in reduced P300 amplitudes for targets following emotionally salient distractors compared to neutral distractors [38–41]. In the same vein, salient distractors themselves elicit higher P300 amplitudes than neutral distractors. Evidence from later ERP components supports the notion of altered attentional processing of food in AN [37, 42]. Interestingly, one study reported an enhanced P300 for high-calorie food compared to low-calorie food in AN [37]. In the other study, the increased P300 and the increased late positive potential in AN was mainly driven by increased motivated processing of low-calorie food [42]. However, these results among later ERPs were presented in prolonged presentation durations and again obtained in passive viewing contexts without competing demands on attention. Consequently, the current findings do not reveal how this bias unfolds under conditions of limited attentional capacity.

Notably, attentional biases to food vary as a function of presentation format [14]. Specifically, when one food stimulus was presented for a prolonged duration, females with AN showed an intensified processing of high-calorie foods, whereas they avoided high-calorie food when attentional disengagement was facilitated [14]. Moreover, late positive ERP components have been shown to be sensitive to different task demands [43]. Therefore, combining RSVP with ERP measures provides a powerful method to concurrently assess early attentional capture and later resource allocation under high perceptual load, both of which may be differentially affected in AN.

The present study aimed to combine the high temporal resolution of ERP measures with the behavioral measure of the attentional blink paradigm to dissect the processing stages, i.e., engagement and disengagement, that underlie food-related attentional biases in AN. Specifically, we employed an RSVP to investigate whether high-calorie food captures and holds attention more strongly in AN than in HC, and whether these effects are reflected in EPN and P300 components. We hypothesized that individuals with AN compared to HC show a biased early attention towards food. Thus, in the RSVP, we expected to see an increased attentional engagement (i.e., a higher accuracy for food targets that follow neutral distractors; H1) [28] and an impaired attentional disengagement (i.e., a lower accuracy of neutral targets that follow food distractors; H2) in the AN group compared to the HC group. Both effects were expected to be higher for high-calorie food compared to low-calorie food. Similarly, we expected enlarged EPN amplitudes (H3) [36] as well as enlarged P300 amplitudes (H4) [42] for food compared to neutral stimuli, an effect which we also expected to be more pronounced for high-calorie than low-calorie food. The study protocol and the hypotheses have been preregistered at the Open Science Framework (OSF; osf.io/zy7rv).

## Methods

### Participants

We included *n* = 33 female adolescents with a diagnosis of AN or EDNOS (only atypical AN) according to the Diagnostic and Statistical Manual of Mental Disorders, 5th Edition (DSM-5) [1] in the AN group and *n* = 36 healthy age-matched female adolescents without a lifetime history of eating disorders in the HC group. Inclusion criteria across groups were age between 12 and 18 years, female sex (assigned at birth), and good or corrected sight. Exclusion criteria across groups were vegan diet, current pregnancy or lactation, high risk of suicide, lifetime psychotic, bipolar, or substance use disorder diagnosis according to DSM-5 and insufficient German language skills. We recruited the AN group at a local inpatient and outpatient clinic and the HC group via the email-list of the local university, posters and flyers. Participants received vouchers for their participation. The present study was conducted in accordance with the ethical code of the World Medical Association (Declaration of Helsinki) and was approved by the institutional review board of the local university (908/2018BO1).

Three females from the HC group were excluded post-measurement due to technical complications (*n* = 1) and due to insufficient segments in the EEG data (*n* = 2) resulting in a final sample of *n* = 33 individuals in the AN group and *n* = 33 individuals in the HC group.

### Material

#### Interviews and questionnaires

AN was diagnosed via the diagnostic items of the Child Eating Disorder Examination (Ch-EDE) [44, 45], an established semi-structured interview. These items were also used to verify the absence of a lifetime history of eating disorders in the HC group. The assessment of comorbid mental disorders was conducted using the Diagnostic Interview for Mental Disorders in Children and Adolescents (Kinder-DIPS) [46]. Ch-EDE and Kinder-DIPS have been demonstrated to be reliable and valid measures [47–49].

Eating disorder pathology was assessed using the Child Eating Disorder Examination Questionnaire (ChEDE-Q) [50], yielding a global score and subscale scores for restrained eating, eating concern, weight concern, and shape concern. Restrained eating was measured using the Restraint subscale of the Dutch Eating Behavior Questionnaire for Children (DEBQ-C) [51, 52]. Depressive symptoms were assessed using the Center for Epidemiologic Studies Depression Scale (CES-D) [53, 54]. Pubertal development was measured with the Pubertal Development Scale (PDS) [55, 56]. All questionnaires demonstrated acceptable to excellent reliability [50, 52, 53, 55].

Before and after the RSVP task, participants provided ratings of their state hunger, mood, and arousal using a 0-100 visual analogue scale (VAS).

#### Rapid serial visual presentation task

The stimulus set included 26 images of high-calorie foods, 26 images of low-calorie foods (all vegetarian), and 84 images of neutral objects (animals), which served as both targets and distractors, as well as 68 images of office supplies used as filler stimuli. Food and neutral stimuli were matched in color, valence, arousal and complexity. All images were selected from an internal database [27]. The stimuli had 550 × 550 pixels and were presented without a background in the middle of a gray screen (RGB 128, 128, 128). Distractors and targets were visually marked with a blue frame, while filler stimuli were presented with a black frame, both 10 pixels wide. The experiment was presented using Presentation software (version 17.0, Neurobehavioral Systems, USA) and was presented on a 1920 × 1200 pixels monitor with a viewing distance from the screen of approximately 70 cm.

**Fig. 1:**
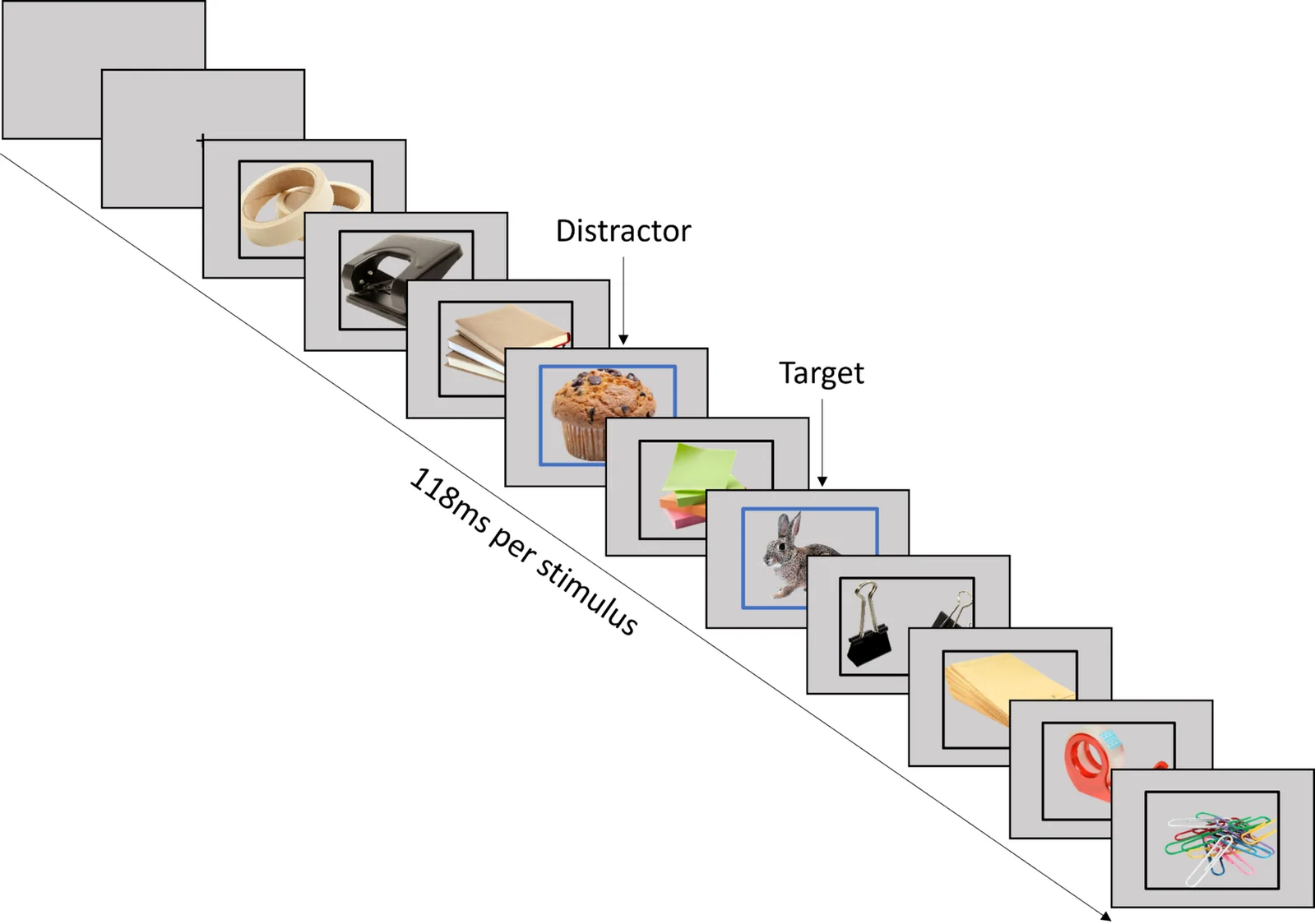
Illustrative Example of a Trial with High-calorie Distractor and Neutral Target. Note. The images depicted here serve illustrative purposes only and were not included in the study

The design of the RSVP task was adapted from the dual-target paradigm described by Neimeijer et al. [57]. Each trial consisted of a rapid stream of stimuli, with each picture presented for 118 ms and no inter-stimulus interval. Every trial contained one distractor and one target stimulus (see Fig. 1). Trials began with three, five or seven filler stimuli preceding a distractor (food or animal image). Each distractor was followed by a filler stimulus, which was then immediately succeeded by a target. Four additional filler stimuli followed the target. At the end of each trial, participants were asked to identify the stimuli they had seen within the first and second blue frames by selecting from eight response options: six possible stimulus items, “I don’t know”, and “none of them”. Each trial was followed by a jittered inter-trial interval (500 to 1,000 ms). In total, 340 trials were presented across five blocks of 68 trials each ensuring that all food and animal stimuli appeared five times (once per block). The RSVP task included five trial types: high-calorie distractor with neutral target, low-calorie distractor with neutral target, neutral distractor with high-calorie target, neutral distractor with low-calorie target, and neutral distractor with neutral target. Both the order of trials and the selection of stimuli within each category were randomized within blocks using a shuffle function. Four practice trials with other neutral stimuli were presented at the beginning of the measurement.

### Procedure

Patients were assessed at the inpatient clinic, while HCs were tested in a laboratory at the local university. Measurements were conducted in comparable settings using the same experimental set-up and identical equipment (duplicate EEG setup) at both locations. Given written informed consent (by participants and their parents), an in-person diagnostic session was conducted and participants’ body weight and height were assessed to calculate Body mass index (BMI) and age- and sex-adjusted BMI z-scores [58]. The experimental session took place individually in a soundproof laboratory under dim lighting conditions. After the application of the EEG setup participants completed the initial set of VAS followed by written instructions. They then proceeded with the practice trials and the RSVP task. After completing the RSVP task, participants filled in a second set of VAS. An additional independent task was conducted as part of the EEG assessment after the RSVP task with a break in between. The results from that study are reported elsewhere [37].

### EEG recording and preprocessing

EEG data was continuously recorded during the RSPV task with BrainVision Recorder 1.20 (Brain Products GmbH, Gliching, Germany) with an actiCHamp amplifier (Brain Products GmbH, Gliching, Germany). The setup included 64 Ag/AgCl active electrodes (actiCAP slim), positioned according to the 10–10 system in an actiCAP slim electrode cap (Brain Products GmbH, Gliching, Germany). Electrode impedances were maintained below 15 kΩ. EEG was recorded with Cz as the online reference and Fpz as the ground electrode, using a sampling rate of 500 Hz and an online filter from 0.1 Hz to 100 Hz [59, 60]. Three electrodes were placed outside the cap: one below the left eye and one on each outer canthus. Together with FP1 (placed above the left eye) these electrodes were used to monitor horizontal and vertical eye movements for later ocular artifact correction.

EEG data were analyzed offline using BrainVision Analyzer 2.1 (Brain Products GmbH, Gliching, Germany). The data were downsampled to 256 Hz, re-referenced to the average reference and filtered with a 0.2 Hz high-pass filter, a 30 Hz low-pass filter and a 50 Hz notch filter. Subsequently, a manual inspection of the raw data was conducted to remove muscle artifacts and other noise. Ocular artifacts caused by horizontal and vertical eye movements were corrected using independent component analysis, followed by an automatic artifact rejection procedure (maximal voltage step = 50 µV/ms, maximal difference of values within 200ms intervals = 200 µV, minimum activity within 100ms intervals < 0.5 µV). Following pre-processing, the EEG signal was segmented into segments ranging from 200ms before to 1,000ms after distractor/target onset. For the analysis of ERPs of the distractors only segments from trials with correct identification of the distractor were used. For the analysis of ERPs of the targets only segments from trials with correct identification of the distractor and the target were used. Participants were excluded from analysis if fewer than 15 artifact-free segments per condition remained. This led to an exclusion of two HC participants for analyses of ERPs of distractors and an additional six participants for analyses of ERPs of targets (*n*_AN_ = 1, *n*_HC_ = 5). Baseline correction was performed using the 200ms pre-stimulus interval. For each participant, condition, and electrode averaged ERPs for distractors and targets were then computed. Mean amplitudes (mean activity; µV) for the EPN were extracted in the time window of 220–340ms after stimulus onset. To analyze the P300 automatic peak detection was carried out preceded by visual inspection in a window from 320–480ms [31].

For statistical analyses, ERP amplitudes were averaged for the respective electrode clusters for each participant and condition. For the EPN of distractors and targets, a medial occipital-parietal cluster was used, consistent with prior studies employing food-related stimuli [27, 36, 37, 61–63]. Accordingly, the EPNs were computed as the average activity across occipital-parietal electrodes, selected based on visual inspection of the grand averages (P7, P5, P3, P1, Pz, P2, P4, P6, P8, PO7, PO3, POz, PO4, PO8, O1, O2, Oz). The P300 of distractors was determined by averaging activity across a central-occipital-parietal electrode cluster (P7, P5, P3, P1, Pz, P2, P4, P6, P8, PO7, PO3, POz, PO4, PO8, CP1, CPz, CP2) and the P300 of targets was computed as the average activity across parietal electrodes (P5, P3, P1, Pz, P2, P4, P6, PO7, PO3, POz, PO4, PO8) based on visual inspection of the grand averages and in line with previous studies [27, 37, 64, 65].

### Data processing and analysis

Due to previous studies [66–68] our power analysis was based on (1–β) = 0.80, *f* = 0.25, and α = 0.05 and conducted with with g*Power [69] indicated a required sample size of 25 participants per group. To account for potential data loss from low-quality EEG recordings, we increased the target sample size to *n* = 32 participants per group.

For the analysis of behavioral effects in the RSVP paradigm only trials with correct identification of the distractor were included. Among them, the percentage of correctly identified targets (i.e., target accuracy) was calculated dependent on *Stimulus Category*, separately for the disengagement condition (varying distractors: high-calorie, low-calorie, neutral; neutral target) and the engagement condition (neutral distractor; varying targets: high-calorie, low-calorie, neutral) for each participant as preregistered [18, 28, 61]. Extreme values defined as an accuracy below three standard deviations from the mean according to group and trial type were excluded. This led to the exclusion of three data points.

The effects of *Group* (AN, HC) and *Stimulus Category* (high-calorie, low-calorie, neutral) on behavioral outcomes and ERPs were determined by linear mixed models (LMMs) with random intercepts for participants using the lme4-package [70] in R. Although target accuracy is a bounded proportion we used LMMs due to minimal ceiling effects (100% accuracy occurring only three times across all participants and conditions), accuracy values not being strongly concentrated at either boundary and a relatively high number of trials per condition [71]. Visual inspection of residuals indicated an acceptable approximation to normal distribution, with only minor deviations at the extreme tails [71]. To test the predictive value of *Group* and *Stimulus Category* and their interaction on ERPs likelihood-ratio tests (LRTs) were performed. For significant effects, post-hoc contrasts with Tukey method for *p*-value adjustment and Kenward-Roger method for degrees of freedom were calculated using the emmeans-package [72]. Age was included as covariate in all models because the age range of 12–18 years encompasses a relatively broad developmental period during adolescence and age-related differences may affect both behavioral performance and electrophysiological processing [73]. As age was not preregistered as a covariate, we also ran all analyses dropping this variable. All results remained the same when age was excluded as a covariate.

Two-sided independent *t*-tests were calculated for group comparisons of the sample characteristics. To determine the effect of *Time* and *Group* on state hunger, mood and arousal, we again performed LMMs. Exploratively, baseline hunger, mood and arousal were each included separately as covariates in all analyses of the dependent variables. Descriptive statistics and model estimators for all significant LMMs are reported in the supplement (Supplementary Table S3-S20).

## Results

### Sample characteristics

The AN group consisted of *n* = 32 patients with restrictive AN and *n* = 1 patient with EDNOS (atypical AN), binge-purge type. Participants’ age ranged from 12 to 18 years and mean age did not differ between groups (*p* = .286; Table 1). As expected, participants in the AN group had a lower BMI and zBMI and higher eating disorder pathology, restrained eating and depressive symptoms (all *p*s < .001) than participants in the HC group (Table 1). Descriptive data and group comparisons of all subscales of ChEDE-Q, DEBQ-C and PDS are reported in Supplementary Table S1 and number and types of comorbidities are reported in Supplementary Table S2.

**Table 1:** Sample characteristics

	AN (*n* = 33) *M* (*SD*)	HC (*n* = 33) *M* (*SD*)	*t*(64)	*p*
Age	14.85 (1.52)	15.29 (1.83)	-1.08	.286
BMI^1^	14.93 (1.32)	20.05 (3.19)	-8.54	< .001
zBMI^1^	-2.69 (0.95)	-0.20 (1.04)	-10.13	< .001
ChEDE-Q	3.22 (1.46)	0.50 (0.57)	9.73^a^	< .001
DEBQ-C Restraint	22.42 (7.97)	4.48 (5.29)	10.44^b^	< .001
CES-D	28.06 (10.80)	10.03 (8.46)	7.40^a^	< .001

Note. Decimal number of exact age in years; Age and gender specific BMI-z-scores [58]. AN = Anorexia nervosa, BMI = Body Mass Index, CES-D = Center for Epidemiologic Studies Depression Scale, ChEDE-Q = Child Eating Disorder Examination Questionnaire, DEBQ-C = Dutch Eating Behavior Questionnaire for Children, HC = healthy controls

^1^BMI percentiles (P) in the AN group: n = 18, P1-P5 n= 11, P6-P15: n = 3, P16-P50: n = 1 and in HCs: P1-P5 n = 5, P6-P15: n = 2, P16-P50: n = 9, P51-P90: n = 15, P91-P97 n = 2 [58]

^a^Based on df = 62, because two participants did not fill in all questions

^b^Based on df = 60, because four participants did not fill in all questions

### Main outcomes

#### Behavioral data

##### Disengagement condition

There were neither significant main effects of *Group* or *Distractor Type*, nor a significant interaction of *Group* × *Distractor Type* (all *p*s ≥ .159) for neutral target accuracy after varying distractors.

##### Engagement condition

**Fig. 2:**
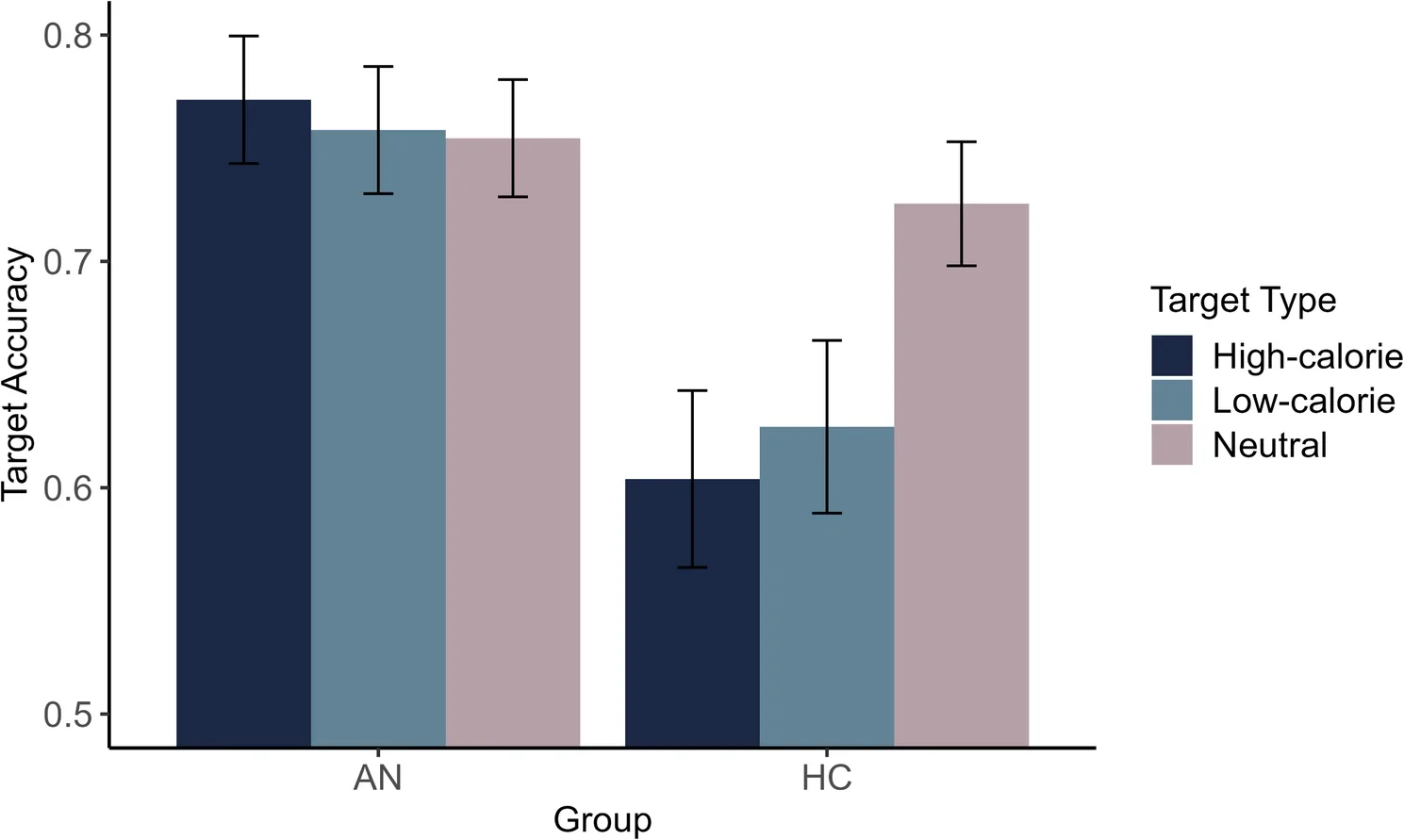
Mean target accuracy after neutral distractors. Note. Depicted are means and standard errors of target accuracy (i.e., percentage of correctly identified targets) after neutral distractors according to *Group* and *Target Type.* Group × Target Type, *χ*^2^ [2] = 25.16, *p* < .001. AN = Anorexia nervosa, *n* = 33, HC = healthy controls, *n* = 33

LRTs indicated a significant interaction of *Group* × *Target Type* (*χ*^2^ [2] = 25.16, *p* < .001; Fig. 2) for target accuracy after neutral distractors. In the AN group, there were no differences in target accuracy depending on stimulus category (all *p*s ≥ .585). In the HC group, target accuracy was lower for both high-calorie and low-calorie targets relative to neutral targets (*t*[133] = -6.16, *p* < .001; *t*[133] = -4.81, *p* < .001, respectively), whereas target accuracy for high-calorie and low-calorie targets was comparable (*p* = .363). Between groups, post hoc contrasts showed a higher target accuracy for high-calorie and low-calorie targets in the AN group than in the HC group (*t*[82.1] = 3.58, *p* < .001; *t*[82.1] = 2.80, *p* = .006, respectively), whereas there was no group difference for neutral targets (*p* = .350).

#### Event-related potentials

##### Distractors

**Fig. 3:**
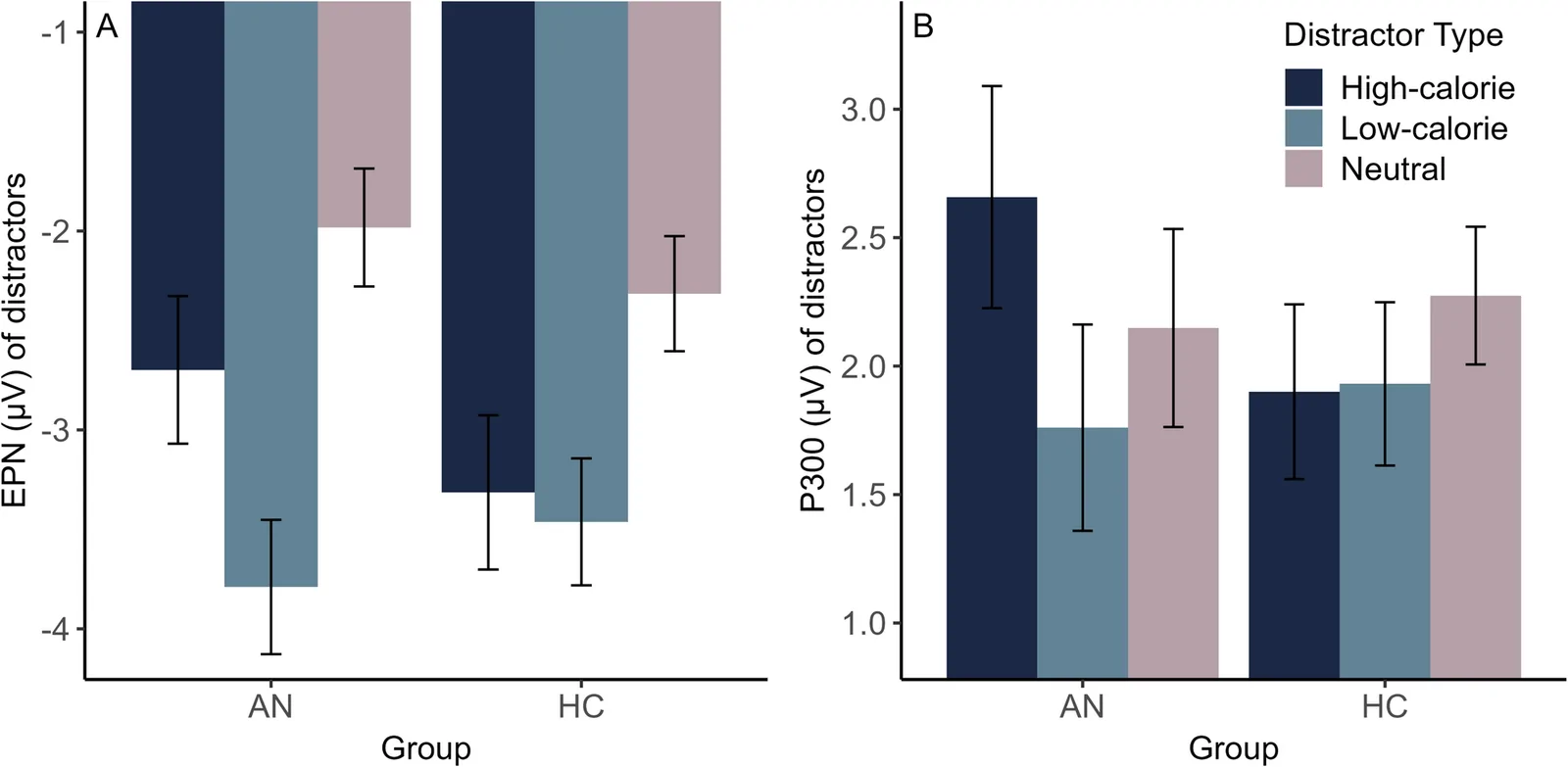
Mean amplitudes of (**A**) EPN and (**B**) P300 of correctly identified distractors. Note. Depicted are mean amplitudes and standard errors of the mean (error bars) according to *Group* and *Distractor Type* of (**A**) EPN mean voltages in the time window 220 ms to 340 ms and (**B**) P300 peaks in the time window of 320–480 ms after distractor stimulus onset. **A** EPN: Group × Distractor Type, *χ*^2^ [2] = 6.67, *p* = .036, (**B**) P300: Group × Distractor Type, *χ*^2^ [2] = 8.88, *p* = .012. AN = Anorexia nervosa, *n* = 33, HC = healthy controls, *n* = 33, EPN = early posterior negativity

**Fig. 4:**
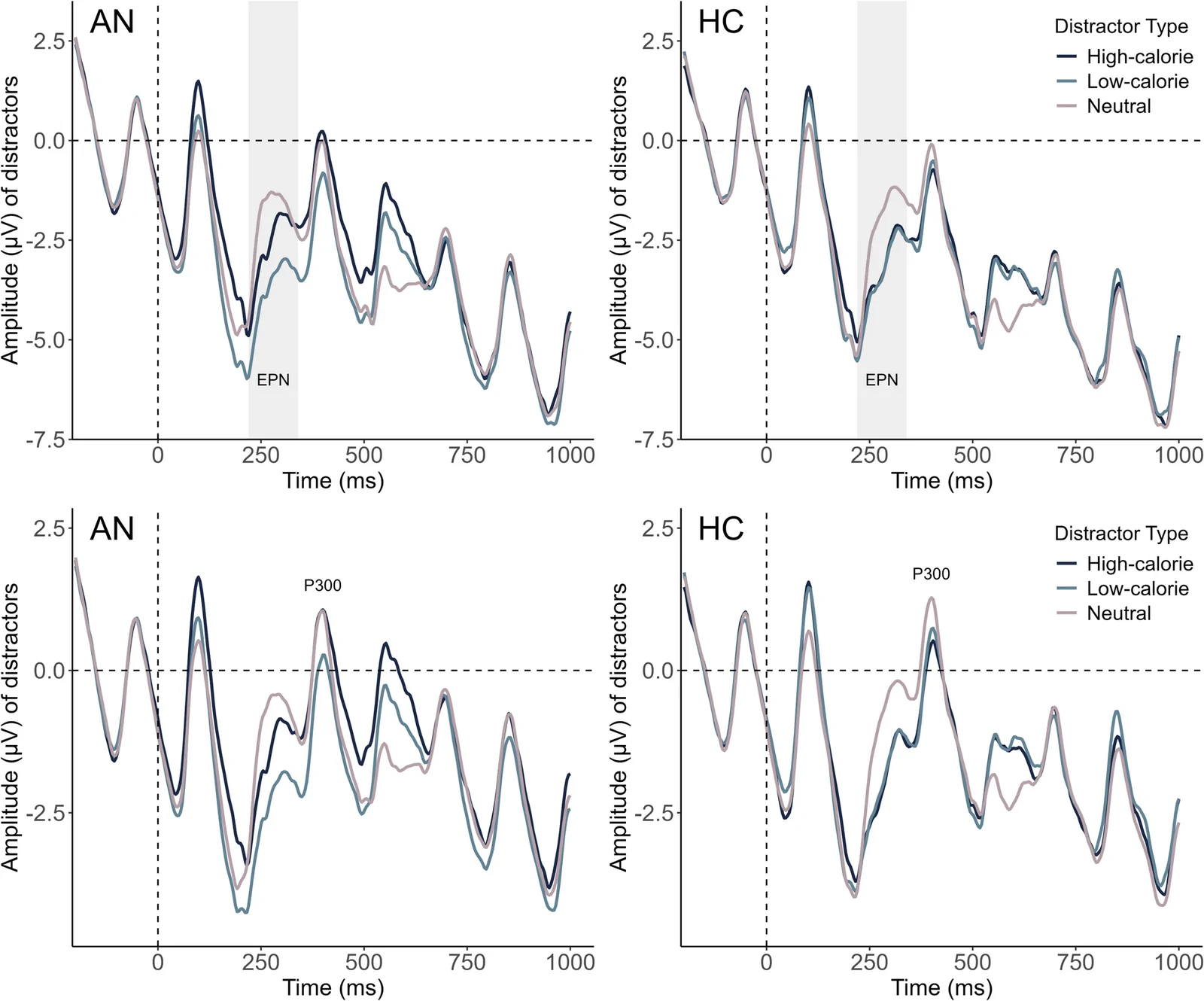
Grand averages of distractors. Note. Depicted are grand averages (µV) for high-calorie, low-calorie and neutral distractors in the AN group (left column) and in HC (right column). Grand averages for EPN (top line) were calculated over occipital parietal electrodes (P7, P5, P3, P1, Pz, P2, P4, P6, P8, PO7, PO3, POz, PO4, PO8, O1, O2, Oz); for P300 (bottom line) we averaged over a central-occipital-parietal cluster (P7, P5, P3, P1, Pz, P2, P4, P6, P8, PO7, PO3, POz, PO4, PO8, CP1, CPz, CP2). The EPN time window (220–340ms) is depicted in light grey. AN = Anorexia nervosa, *n* = 33, HC = healthy controls, *n* = 33, EPN = early posterior negativity. Note the varying scaling of the axes

For the EPN elicited by distractors, LRTs indicated a significant interaction of *Group* × *Distractor Type* (*χ*^2^ [2] = 6.67, *p* = .036; Fig. 3A and 4). Post-hoc contrasts showed that in the AN group, low-calorie food elicited higher EPN amplitudes than high-calorie food (*t*[136] = 4.11, *p* < .001) and than neutral stimuli (*t*[136] = -6.80, *p* < .001). Additionally, high-calorie food elicited higher EPN amplitudes than neutral stimuli (*t*[136] = -2.69, *p* < .022). In the HC group, EPN amplitudes of high- and low-calorie food did not differ (*t*[136] = 0.56, *p* = .843), but both stimulus categories elicited higher EPN amplitudes than neutral stimuli (*t*[136] = -3.76, *p* < .001; *t*[136] = -4.32, *p* < .001, respectively). Between groups, post-hoc contrasts showed no significant differences in the EPN of distractors (all *p*s ≥ .166).

For the P300 elicited by distractors, LRTs indicated a significant interaction of *Group* × *Distractor Type* (*χ*^2^ [2] = 8.88, *p* = .012; Fig. 3B and 4). Post-hoc contrasts showed that in the AN group, high-calorie food elicited higher P300 amplitudes than low-calorie food (*t*[136] = 3.618, *p* = .001). There were no other significant post-hoc contrasts neither in the AN group nor and in the HC group. There were no further significant between group differences (all *p*s ≥ .103).

##### Targets: disengagement condition

**Fig. 5:**
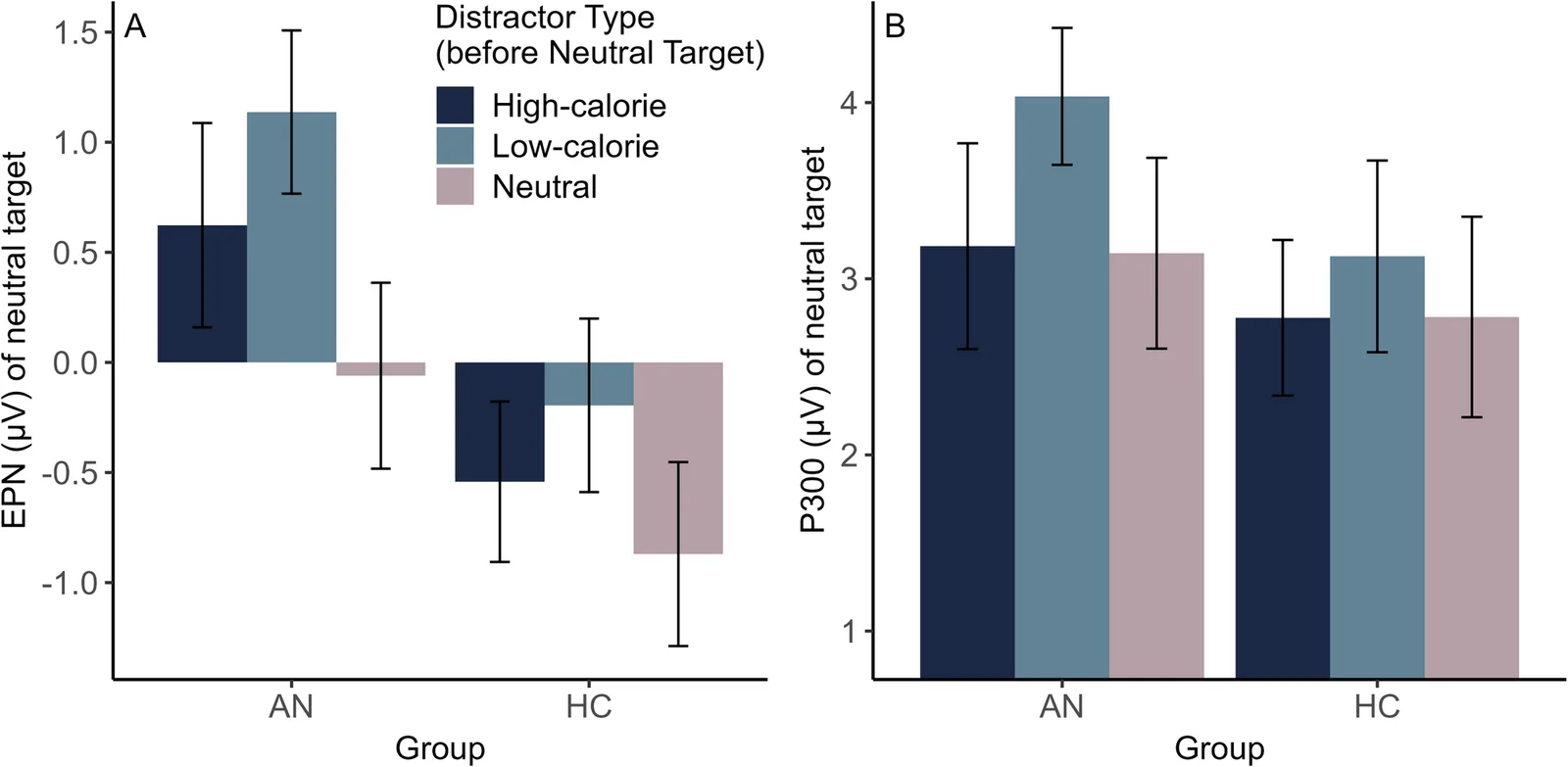
Mean amplitudes of (**A**) EPN and (**B**) P300 of correctly identified neutral targets. Note. Depicted are mean amplitudes and standard errors of the mean (error bars) of neutral targets according to *Group* and *Distractor Type* of (**A**) EPN mean voltages in the time window 220 ms to 340 ms and (**B**) P300 peaks in the time window of 320–480 ms after target stimulus onset. **A** EPN: Group + Distractor Type, *χ*^2^ [2] = 5.62, *p* = .018, (B) P300: Distractor Type, *χ*^2^ [2] = 8.61, *p* = .014. AN = Anorexia nervosa, *n* = 32, HC = healthy controls, *n* = 28, EPN = early posterior negativity

**Fig. 6:**
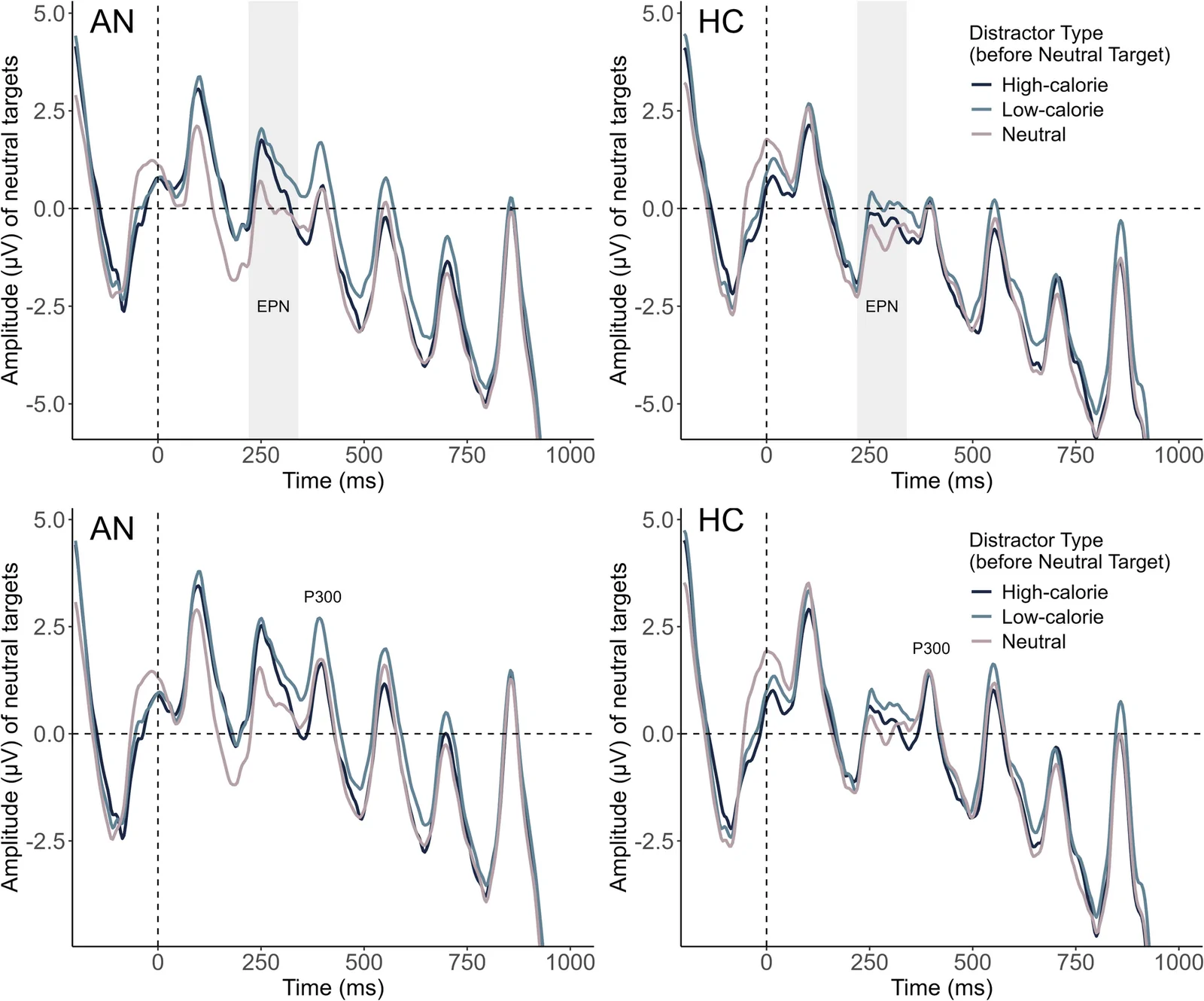
Grand averages of neutral targets. Note. Depicted are grand averages (µV) of neutral targets following high-calorie, low-calorie and neutral distractors in the AN group (left column) and in HC (right column). Grand averages for EPN (top line) were calculated over occipital parietal electrodes (P7, P5, P3, P1, Pz, P2, P4, P6, P8, PO7, PO3, POz, PO4, PO8, O1, O2, Oz); for P300 (bottom line) we averaged over a parietal cluster (P5, P3, P1, Pz, P2, P4, P6, PO7, PO3, POz, PO4, PO8). The EPN time window (220–340ms) is depicted in light grey. AN = Anorexia nervosa, *n* = 32, HC = healthy controls, *n* = 28, EPN = early posterior negativity. Note the varying scaling of the axes

For the EPN elicited by neutral targets after varying distractors, LRTs indicated significant main effects of *Group* and *Distractor Type* (*χ*^2^ [1] = 5.62, *p* = .018; Fig. 5A and 6). Irrespective of group membership, neutral targets following neutral distractors elicited higher EPN amplitudes compared to neutral targets following high-calorie distractors and compared to neutral targets following low-calorie food distractors (*t*[122] = 2.61, *p* = .027; *t*[122] = 4.81, *p* < .001, respectively). EPN amplitudes of neutral targets following high-calorie food distractors and of neutral targets following low-calorie food distractors were comparable (*p* = .075). Additionally, EPN amplitudes of neutral targets in the AN group were lower than in the HC group (*t* [2] = 2.37, *p* = .021), regardless of distractor type.

For the P300 elicited by neutral targets after varying distractors, LRTs indicated a significant main effect of *Distractor Type* (*χ*^2^ [2] = 8.61, *p* = .014; Fig. 5B and 6). Irrespective of group membership, neutral targets following low-calorie food distractors elicited higher P300 amplitudes than neutral targets following either high-calorie food distractors or neutral distractors (*t*[122] = -2.53, *p* = .034; *t*[122] = 2.60, *p* = .028, respectively).

##### Targets: engagement condition

**Fig. 7:**
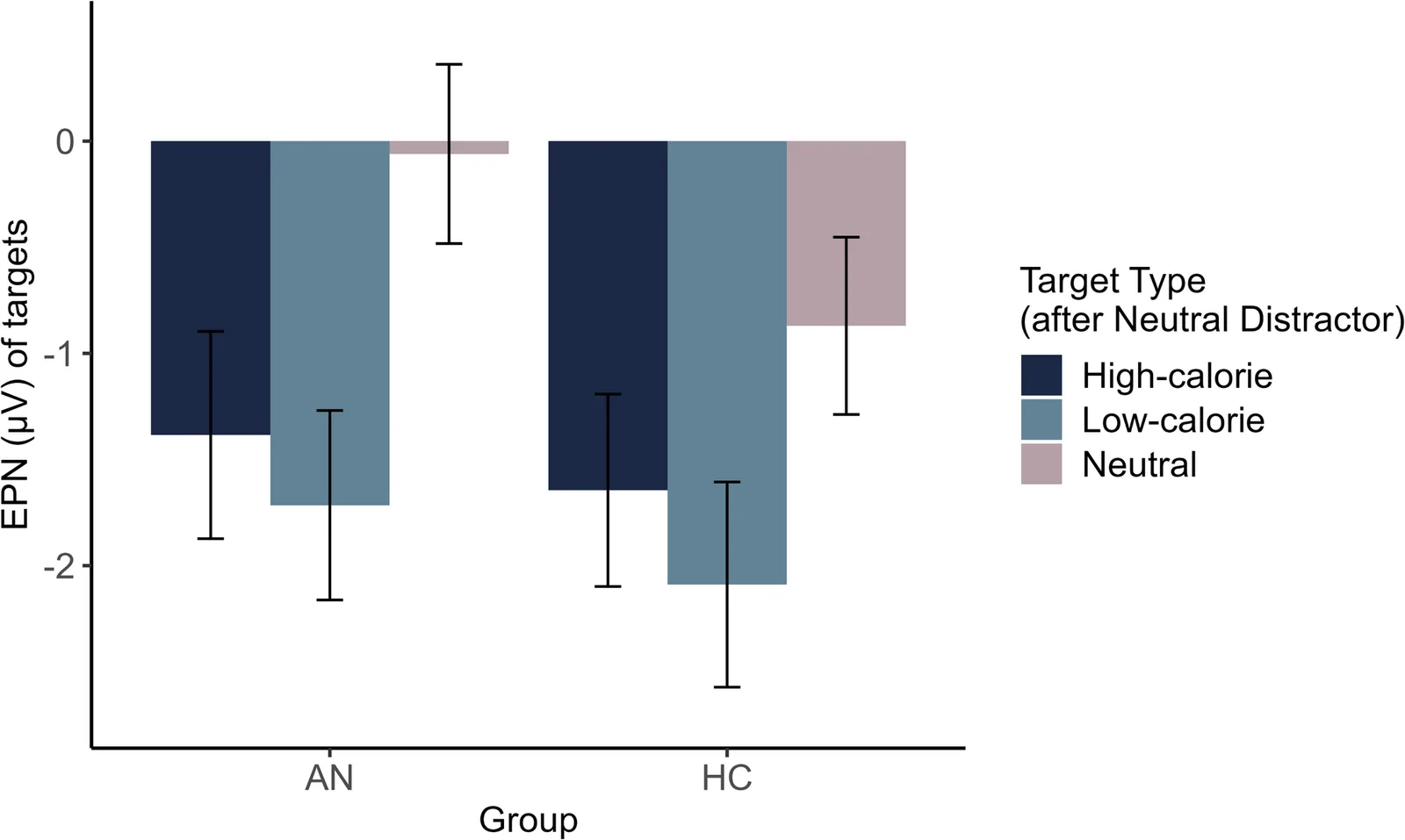
Mean amplitudes of EPN of correctly identified targets after neutral distractors. Note. Depicted are mean amplitudes and standard errors of the mean (error bars) of targets after neutral distractors according to *Group* and *Target Type* of EPN mean voltages in the time window 220 ms to 340 ms after target stimulus onset. Target Type (*χ*^2^ [2] = 56.19, *p* < .001. AN = Anorexia nervosa, *n* = 32, HC = healthy controls, *n* = 28, EPN = early posterior negativity

**Fig. 8:**
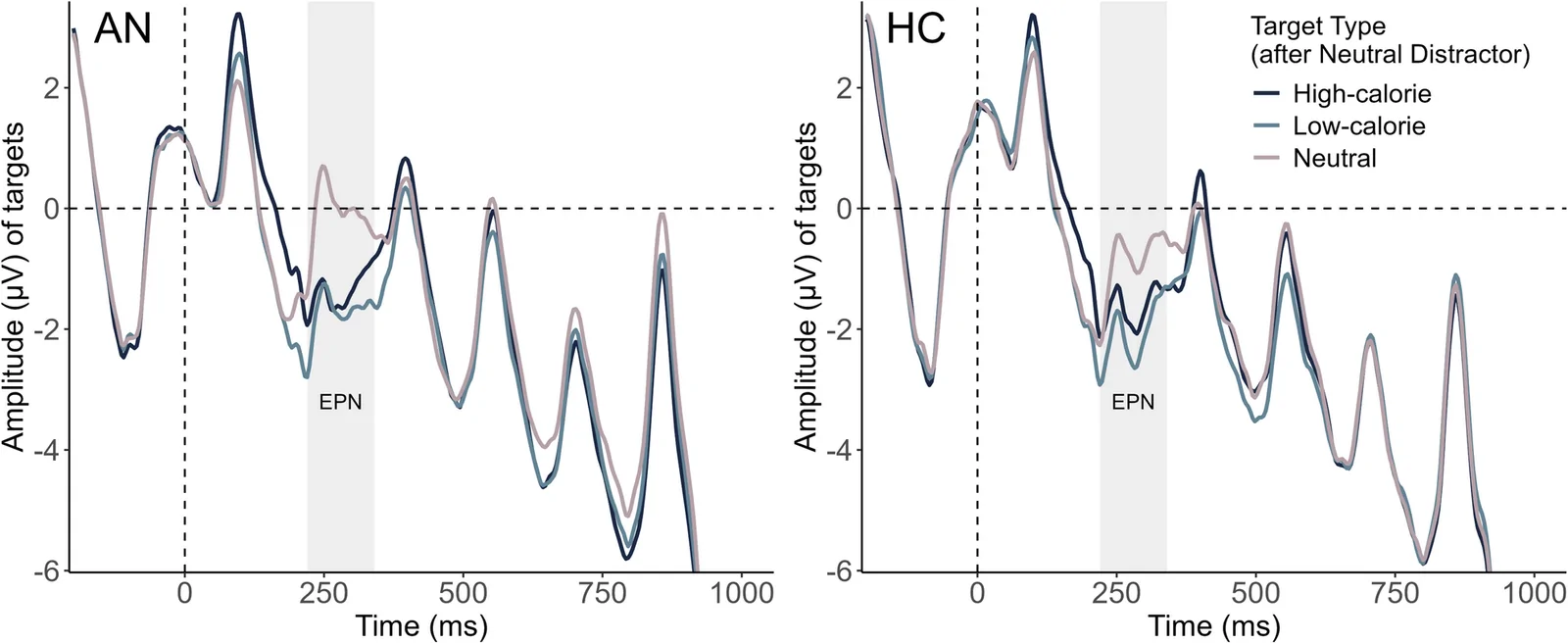
Grand Averages of targets after neutral distractors. Note. Depicted are grand averages (µV) of high-calorie, low-calorie and neutral targets following neutral distractors in the AN group (left) and in HC (right) for the EPN. Grand averages were calculated over occipital parietal electrodes (P7, P5, P3, P1, Pz, P2, P4, P6, P8, PO7, PO3, POz, PO4, PO8, O1, O2, Oz). The EPN time window (220–340ms) is depicted in light grey. AN = Anorexia nervosa, *n* = 32, HC = healthy controls, *n* = 28, EPN = early posterior negativity

For the EPN elicited by targets after neutral distractors, LRTs indicated a significant main effect of the *Target Type* (*χ*^2^ [2] = 56.19, *p* < .001; Fig. 7 and 8). Irrespective of group membership, high- and low-calorie food targets elicited higher EPN amplitudes than neutral targets (*t*[122] = -5.96, *p* < .001; *t*[122] = -8.10, *p* < .001, respectively). EPN amplitudes of high- and low-calorie food targets did not differ (*p* = .086).

For the P300 elicited by targets after neutral distractors, LRTs indicated no interaction effect of *Group* × *Target Type* and no main effects of *Group* or *Target Type* (all *p*s ≥ .099).

### Self-report state measures

For hunger, LRTs indicated significant main effects of *Time* and *Group* (*χ*^2^ [1] = 8.35, *p* = .004). Hunger increased during the task (*t* [66] = 3.91, *p* < .001) and was higher in the HC group than in the AN group (*t*[67.1] = -2.94, *p* = .005).

For mood, LRTs indicated a significant interaction of *Time* × *Group* (*χ*^2^ [1] = 4.19, *p* = .041). Before and after the task, mood was better in the HC group than in the AN group (*t*[105] = -4.21, *p* < .001; *t*[105] = -2.21, *p* = .029, respectively). Additionally, there was a significant mood decline during the task across groups which was more pronounced in the HC group (*t*[67.1] = -6.53, *p* < .001) than in the AN group (*t*[67.1] = -3.71, *p* < .001).

For arousal, LRTs indicated significant main effects of *Time* and *Group (χ*^2^ [1] = 9.18, *p* = .002). Arousal decreased during the task (*t* [66] = 2.58, *p* = .012) and was higher in the AN group than in the HC group (*t*[67.1] = 3.09, *p* = .003).

Including baseline hunger or arousal as a covariate did not change any of the main results. Similarly, the results remained unchanged when baseline mood was included as a covariate, with one exception: For the EPN elicited by neutral targets after varying distractors, LRTs indicated a significant main effect of *Distractor Type* (*χ*^2^ [2] = 20.10, *p* < .001). In contrast to the original analysis, in which the model including main effects of *Distractor Type* and *Group* was significant, adding the main effect of *Group* to the model only resulted in a marginal improvement in model fit (*χ*² [1] = 3.65, *p* = .057).

## Discussion

The present study combined behavioral measures (target accuracy) and ERPs (EPN, P300) to investigate the temporal dynamics of food-related attentional bias in adolescents with AN. Specifically, we employed an attentional blink paradigm via an RSVP task to test whether food captures and sustains attention more strongly in AN than in HC.

As hypothesized, behavioral results suggest an increased attentional *engagement* for food in AN. This is demonstrated in a higher target accuracy for high- and low-calorie food that followed neutral distractors in the AN group compared to the HC group. The accuracy of neutral targets did not differ between the groups. Contrary to our hypothesis, however, our findings revealed no evidence of impaired attentional *disengagement* from food distractors. This is demonstrated by the comparable target accuracy across distractor types and groups.

To our knowledge, this is the first study to demonstrate facilitated attentional *engagement* for food stimuli in AN using an attentional blink paradigm. An RSVP study in restrained eaters found no enhanced engagement for food relative to neutral stimuli [57], although the use of a non-clinical student sample limits comparability with our clinical AN sample. Facilitated engagement to food has, however, been demonstrated in individuals with binge eating disorder relative to overweight controls [74], and similar engagement effects for emotionally salient stimuli have been observed in other mental disorders, such as spider phobia [21]. Overall, these effects are thought to reflect enhanced bottom-up processing rather than capacity-limited top-down mechanisms [18], suggesting that food stimuli receive prioritized attentional processing in AN. This effect was independent of caloric content, indicating that both high and low-calorie food is motivationally salient. Note however, that their salience may arise from different mechanisms: high-calorie foods may be prioritized because of their negative emotional valence, whereas low-calorie foods may derive salience from positive associations. Consistent with this interpretation, patients with AN in previous studies preferentially attended to low-calorie food while avoiding high-calorie food when both categories are presented simultaneously [14] and report reduced wanting for high-calorie but increased wanting for low-calorie foods compared to controls [75]. Together, these findings suggest that although both food categories capture attention in AN, they may do so for distinct motivational reasons.

In contrast to our findings, previous studies have reported impaired *disengagement* from food in AN. Neimeijer et al. [28] demonstrated reduced accuracy for neutral targets following food distractors in AN, and restrained eaters similarly showed disengagement difficulties from food distractors [57]. A possible explanation for this discrepancy is a ceiling effect: In our study, neutral stimuli (animals) were matched to food stimuli in valence and arousal, whereas previous studies used more heterogeneous disorder-neutral images [28, 57]. Because the attentional blink is reduced for highly arousing and emotionally salient targets [76], the high detection rates in our neutral targets may have masked potential disengagement effects. Highly salient targets rely less on limited attentional resources and are therefore less affected by preceding distractors [18]. At the same time, the enhanced accuracy for food targets despite equally salient neutral (animal) distractors further emphasizes the privileged attentional engagement elicited by food in adolescents with AN.

Taking a closer look at the neural temporal processing of distractors, ERP results differentiated between early and later stages of attentional processing. The EPN indicated enhanced processing of food distractors in patients with AN, but with reduced amplitudes to high-calorie compared to low-calorie food. Thus, despite the overall heightened salience of food reflected in the behavioral engagement effects, high-calorie food may be subject to an early automatic avoidance mechanism. This pattern is consistent with recent findings in a free picture viewing paradigm [37], but contrasts with earlier ERP studies reporting enhanced early processing of high-calorie food in AN [36, 66, 77]. However, behavioral evidence in AN supports reduced automatic approach tendencies towards high-calorie food [78–80] and increased response inhibition following the exposure to high-calorie food [81]. This early avoidance may reflect the reduced incentive value of high-calorie food, indicating a loss of its appetitive properties [82]. Consistent with this interpretation, patients with AN show reduced activation in reward-related brain regions in response to food [83] and diminished sensitivity to food palatability [84].

Notably, the P300 showed the opposite pattern to the EPN. Patients with AN exhibited larger P300 amplitudes for high-calorie than low-calorie food distractors, whereas healthy controls showed no differentiation between food categories. Consistent with previous studies, this suggests enhanced motivated, top-down processing of high-calorie food in AN [14, 37, 42, 77, 85]. This enhanced processing may reflect the increased motivational relevance of high-calorie food driven by its negative emotional salience. Indeed, patients with AN evaluate high-calorie food more negatively and reported anxiety and disgust in response to such stimuli [14, 77, 86–88]. In line with this, enhanced P300 amplitudes have consistently been linked to the processing of threatening and aversive stimuli [89, 90].

Furthermore, early automatic processing of neutral targets, as indexed by reduced EPN amplitudes, was attenuated following food compared to neutral distractors in both groups. This suggests that automatic, bottom-up processing of targets is diminished when attentional resources are already bound to preceding food stimuli. Although we expected this effect to be more pronounced in patients with AN, it was comparable across groups, indicating a more general effect of food on early attentional processing. Similarly, motivated, top-down processing of neutral targets, reflected by the P300, was reduced following high-calorie and neutral distractors, but not low-calorie distractors, irrespective of group membership. This pattern suggests limited capacity for target consolidation when processing resources are occupied by a highly salient distractor (i.e., high-calorie food or animal). Importantly, these neural interference effects were not mirrored in behavioral performance, as target recognition accuracy remained comparable across distractor type. In line with Anderson [18], emotionally salient stimuli benefit from enhanced bottom-up encoding and are therefore less dependent on limited processing resources to reach awareness. Thus, although salient distractors reduce the neural resources available for subsequent targets, behavior can remain intact because salient targets (i.e., animals) still gain access to awareness. Nevertheless, the ERP results indicate that this preserved performance comes at the cost of reduced neural processing of targets following food distractors.

Note that for both high-calorie and low-calorie food targets following neutral distractors, early automatic engagement, indexed by larger EPN amplitudes, was enhanced relative to neutral targets across groups, with no AN-specific effects. In contrast, later motivated top-down processing, reflected by the P300, did not differ between food and neutral targets in either group. Thus, the AN-specific ERP effects observed for distractors, i.e., reduced early processing but enhanced later processing of high-calorie food, were absent during target processing. One possible explanation is that the ongoing processing of neutral distractors in working memory reduces the availability of inhibitory control mechanisms [91], thereby attenuating the early avoidance of high-calorie food in AN. Consistent with this account, the attentional blink has been proposed to reflect a bottleneck in working memory consolidation that primarily affects post-perceptual processing: targets may be perceptually encoded but fail to reach awareness when consolidation resources are occupied [92]. Although no study has directly manipulated cognitive load in attentional bias paradigms in AN, attentional interference by negative valenced stimuli is reduced under increased working-memory load in healthy individuals [93]. Together, these findings suggest that attentional biases are not entirely autonomous but depend, at least in part, on the availability of attentional resources. Accordingly, the processing of high-calorie food in AN may be particularly sensitive to concurrent cognitive demands.

This study has several methodological strengths, including a well-powered sample, the combination of an RSVP paradigm with ERP recordings, and the inclusion of high-calorie and low-calorie food stimuli. Nevertheless, several limitations should be considered. First, both distractors and targets were visually highlighted and had to be identified at the end of each trial, rendering the distractor task-relevant. This design served two purposes: it enabled the assessment of attentional engagement by allowing competition between neutral distractor and target processing and it ensured successful distractor detection, which was essential for the ERP analyses because only correctly identified distractors were included. However, this approach precludes conclusions about the automatic capture of attention by task-irrelevant food stimuli. Because participants were required to process and report the distractors, attentional allocation may have been influenced by task demands and strategic processing. Future studies should therefore employ single-target RSVP paradigms in which food stimuli serve as task-irrelevant distractors. Such designs would allow a more direct examination of whether food captures attention automatically, independent of current task goals.

Second, disorder-neutral stimuli (i.e., animals) were matched to the food stimuli on valence and arousal ratings obtained from healthy individuals. This approach was intended to maximize sensitivity for detecting differential processing between patients with AN and healthy controls, given that food stimuli generally elicit stronger behavioral and electrophysiological responses than emotionally neutral stimuli in healthy individuals [61, 94, 95]. However, because the animal stimuli were matched to the food stimuli on valence and arousal, they may themselves have been relatively salient and should therefore not be considered an emotionally neutral baseline. Moreover, as discussed above, matching stimuli on valence and arousal may have obscured potential disengagement effects. Future studies should therefore include both emotionally neutral and valence-matched non-food stimuli to disentangle food-specific attentional biases from effects driven by emotional salience more generally.

Third, targets occurred at a fixed interval of two positions following the distractor. Therefore, participants may have developed expectations regarding the target’s appearance. Consequently, we did not interpret absolute accuracy rates but instead focused on the relative differences between food and neutral distractors. To address this limitation, future studies should jitter the distractor-target interval and include longer lags (e.g., eight positions), thereby enabling the interpretation of absolute accuracy.

Fourth, our sample predominantly comprised patients with restrictive AN and only one patient with (atypical) AN, binge-purge type. Therefore, our findings does not allow for conclusions regarding potential differences between AN subtypes. As the existing literature among food-related attentional processes in AN has predominantly included patients with restrictive AN [37, 66, 77–79, 81], future studies should aim to include sufficiently large samples of both AN subtypes to allow for a systematic investigation of potential similarities and differences in those attentional processes.

## Conclusions

The present findings suggest that temporal attentional biases for high-calorie food in AN are not a unitary phenomenon but reflect a dynamic interplay between early automatic salience and enhanced later, motivated processing. While the reduced early automatic salience reflects a diminished incentive value of food, the enhanced late-stage processing may be driven by negative emotional salience. Importantly, this AN-specific pattern was contingent on attentional resource availability, as it was no longer evident for target processing, suggesting that these biases are not entirely autonomous but depend, at least in part, on the availability of attentional resources. Whether this dependence extends to naturalistic contexts involving higher cognitive or emotional demand remains an open question, as attentional load was not directly manipulated in the present study. However, if confirmed by studies that experimentally vary load, such resource-dependence could inform the timing and design of attention- and reward-based interventions for high-calorie processing in AN. In addition, the differentiated ERP responses to high-calorie food may serve as a candidate biomarker of treatment response in AN and could complement symptom-based measures, which capture overall symptomatic change but may not distinguish between the underlying mechanisms driving it. However, longitudinal studies are needed to test whether these components shift with treatment and predict clinical outcome.

## Supplementary information

Below is the link to the electronic supplementary material.

Supplementary Material 1

## Data Availability

The datasets analyzed during the current study are available in the OSF repository (https://osf.io/fy4pn).

## Abbreviations

AN: Anorexia nervosa
BMI: Body mass index
CES-D: Center for Epidemiologic Studies Depression Scale
ChEDE-Q: Child Eating Disorder Examination Questionnaire
DEBQ-C: Dutch Eating Behavior Questionnaire for Children
EEG: Electroencephalography
EPN: Early posterior negativity
ERP: Event-related potential
HC: Healthy controls
LMM: Linear mixed model
LRT: Likelihood-ratio test
OSF: Open Science Framework
PDS: Pubertal Development Scale
RSVP: Rapid serial visual presentation
VAS: Visual analogue scales
