# Supplementary material for: Temporal attentional bias to food stimuli in adolescent anorexia nervosa – an ERP study
Source: J Eat Disord. 2026 Oct 22;14:247. doi: 10.1186/s40337-026-01800-w (PMC13688865; doi:10.1186/s40337-026-01800-w)
Supplement: Supplementary file 1 [file 40337_2026_1800_MOESM1_ESM.docx]

**Supplement**

**Temporal Attentional Bias to Food Stimuli in Adolescent Anorexia nervosa – An ERP study**

***Table of contents***

[**Supplementary Table S1**	2](#_Toc235700129)

[**Supplementary Table S2**	3](#_Toc235700130)

[**Supplementary Table S3**	4](#_Toc235700131)

[**Supplementary Table S4**	5](#_Toc235700132)

[**Supplementary Table S5**	5](#_Toc235700133)

[**Supplementary Table S6**	6](#_Toc235700134)

[**Supplementary Table S7**	6](#_Toc235700135)

[**Supplementary Table S8**	7](#_Toc235700136)

[**Supplementary Table S9**	7](#_Toc235700137)

[**Supplementary Table S10**	8](#_Toc235700138)

[**Supplementary Table S11**	8](#_Toc235700139)

[**Supplementary Table S12**	9](#_Toc235700140)

[**Supplementary Table S13**	9](#_Toc235700141)

[**Supplementary Table S14**	10](#_Toc235700142)

[**Supplementary Table S15**	10](#_Toc235700143)

[**Supplementary Table S16**	11](#_Toc235700144)

[**Supplementary Table S17**	12](#_Toc235700145)

[**Supplementary Table S18**	13](#_Toc235700146)

[**Supplementary Table S19**	13](#_Toc235700147)

[**Supplementary Table S20**	13](#_Toc235700148)

[**References**	14](#_Toc235700149)

# **Supplementary Table S1**

*Child Eating Disorder Examination Questionnaire, Dutch Eating Behavior Questionnaire for Children and Pubertal Development Scale scores.*

|  | AN (*n* = 33)  *M* (*SD*) | HC (*n* = 31)  *M* (*SD*) | *t*(62)^a^ | *p* |
| --- | --- | --- | --- | --- |
| ChEDE-Q |  |  |  |  |
| Restraint | 2.90 (1.69) | 0.23 (0.41) | 8.43 | < .001 |
| Eating Concern | 2.60 (1.33) | 0.35 (0.58) | 8.60 | < .001 |
| Weight Concern | 3.46 (1.61) | 0.47 (0.60) | 9.69 | < .001 |
| Shape Concern | 3.96 (1.60) | 0.93 (1.03) | 8.97 | < .001 |
| DEBQ-C |  |  |  |  |
| Restraint | 22.42 (7.97) | 4.48 (5.29) | 10.44^b^ | < .001 |
| Emotional Eating | 4.39 (4.70) | 6.74 (6.11) | -1.70^b^ | .094 |
| External Eating | 11.02 (6.55) | 16.71 (5.64) | -3.67^b^ | < .001 |
| PDS | 9.73 (1.96) | 10.03 (2.65) | -0.53^c^ | .600 |

*Note*. AN = Anorexia nervosa, ChEDE-Q = Child Eating Disorder Examination Questionnaire [1], DEBQ-C = Dutch Eating Behavior Questionnaire for Children [2], HC = healthy controls, PDS = Pubertal Development Scale.

^a^ *df* = 62, because two participants did not fill in all questions, ^b^ Based on *df* = 60, because four participants did not fill in all questions, ^c^ Based on *df* = 63, because one participant did not fill in all questions.

# **Supplementary Table S2**

*Number and types of current comorbidities.*

|  | AN | HC |
| --- | --- | --- |
| Number of comorbidities |  |  |
| One | 10 | 3 |
| Two | 3 | 0 |
| More than two | 2 | 0 |
| Disorder category |  |  |
| Affective disorders | 12 | 0 |
| Anxiety disorders | 6 | 3 |
| Insomnia | 2 | 0 |
| Obsessive compulsive disorder | 1 | 0 |

*Note.* AN = Anorexia nervosa, *n* = 33; HC = healthy controls, *n* = 33.

# **Supplementary Table S3**

*Descriptive statistics of* *target accuracy of the disengagement condition.*

| Distractor type | AN  *M* (*SD*) | HC  *M* (*SD*) |
| --- | --- | --- |
| High-calorie | 0.74 (0.18) | 0.67 (0.26) |
| Low-calorie | 0.76 (0.19) | 0.69 (0.23) |
| Neutral | 0.75 (0.15) ^a^ | 0.73 (0.15)^a^ |

*Note.* Mean target accuracy (i.e., percentage of correctly identified targets) and standard deviations according to *Group* and *Distractor Type*. AN = Anorexia nervosa, *n* = 33, HC = healthy controls, *n* = 33. ^a^ Based on *n* = 32, because of the exclusion of an extreme value.

# **Supplementary Table S4**

*Descriptive statistics of target accuracy of the engagement condition.*

| Target Type | AN  *M* (*SD*) | HC  *M* (*SD*) |
| --- | --- | --- |
| High-calorie | 0.77 (0.16)^a^ | 0.60 (0.22) |
| Low-calorie | 0.76 (0.16)^a^ | 0.63 (0.22) |
| Neutral | 0.75 (0.15) ^a^ | 0.73 (0.15)^a^ |

*Note.* Mean target accuracy (i.e., percentage of correctly identified targets) and standard deviations after neutral distractors according to *Group* and *Target Type*. AN = Anorexia nervosa, *n* = 33, HC = healthy controls, *n* = 33. ^a^ Based on *n* = 32, because of the exclusion of an extreme value.

# **Supplementary Table S5**

*Results of the linear mixed model testing the influence of group and target type on target accuracy after neutral distractors.*

|  | Estimate | *SE* | *df* | *t* | *p* |
| --- | --- | --- | --- | --- | --- |
| Intercept | 0.82 | 0.19 | 64.83 | 4.23 | < .001 |
| Target Type: low-calorie | -0.01 | 0.02 | 128.54 | -0.79 | .430 |
| Target Type: neutral | -0.02 | 0.02 | 128.54 | -1.00 | .317 |
| Group: HC | -0.17 | 0.05 | 77.81 | -3.67 | < .001 |
| Age | 0.00 | 0.01 | 64.50 | -0.25 | .805 |
| Target Type: low-calorie × Group: HC | 0.04 | 0.02 | 128.54 | 1.54 | .127 |
| Target Type: neutral × Group: HC | 0.12 | 0.02 | 128.61 | 5.12 | < .001 |

Note. Target accuracy is defined as the percentage of correctly identified targets. HC = healthy controls. Age was included as a covariate.

# **Supplementary Table S6**

*Descriptive statistics of early posterior negativity of distractors.*

| Distractor Type | AN  *M* (*SD*) | HC  *M* (*SD*) |
| --- | --- | --- |
| High-calorie | -2.70 (2.13) | -3.31 (2.22) |
| Low-calorie | -3.79 (1.94) | -3.46 (1.83) |
| Neutral | -1.98 (1.70) | -2.32 (1.66) |

*Note.* Mean amplitudes and standard deviations of early posterior negativity mean voltages (μV) in the time window of 220 ms to 340 ms after stimulus onset for correctly identified distractors according to *Group* and *Distractor Type*. AN = Anorexia nervosa, *n* = 33, HC = healthy controls, *n* = 33.

# **Supplementary Table S7**

*Results of the linear mixed model testing the influence of group and distractor type on early posterior negativity amplitudes of distractors.*

|  | Estimate | *SE* | *df* | *t* | *p* |
| --- | --- | --- | --- | --- | --- |
| Intercept | -4.42 | 1.88 | 66.86 | -2.35 | .022 |
| Distractor Type: low-calorie | -1.09 | 0.26 | 132.00 | -4.17 | < .001 |
| Distractor Type: neutral | 0.72 | 0.26 | 132.00 | 2.73 | .007 |
| Group: HC | -0.67 | 0.47 | 101.79 | -1.43 | .157 |
| Age | 0.12 | 0.12 | 66.00 | 0.93 | .357 |
| Distractor Type: low-calorie × Group: HC | 0.94 | 0.37 | 132.00 | 2.55 | .012 |
| Distractor Type: neutral × Group: HC | 0.28 | 0.37 | 132.00 | 0.76 | .446 |

Note. HC = healthy controls. Age was included as a covariate.

# **Supplementary Table S8**

*Descriptive statistics of the P300 of distractors.*

| Distractor Type | AN  *M* (*SD*) | HC  *M* (*SD*) |
| --- | --- | --- |
| High-calorie | 2.66 (2.48) | 1.90 (1.95) |
| Low-calorie | 1.76 (2.31) | 1.93 (1.83) |
| Neutral | 2.15 (2.21) | 2.27 (1.54) |

*Note.* Mean amplitudes and standard deviations of P300 peaks (μV) in the time window of 320 ms – 480 ms after stimulus onset for correctly identified distractors according to *Group* and *Distractor Type*. AN = Anorexia nervosa, *n* = 33, HC = healthy controls, *n* = 33.

# **Supplementary Table S9**

*Results of the linear mixed model testing the influence of group and distractor type on P300 of distractors.*

|  | Estimate | *SE* | *df* | *t* | *p* |
| --- | --- | --- | --- | --- | --- |
| Intercept | 5.26 | 2.08 | 66.61 | 2.53 | .014 |
| Distractor Type: low-calorie | -0.90 | 0.24 | 132.00 | -3.67 | < .001 |
| Distractor Type: neutral | -0.51 | 0.24 | 132.00 | -2.09 | .039 |
| Group: HC | -0.68 | 0.50 | 91.38 | -1.35 | .180 |
| Age | -0.18 | 0.14 | 66.00 | -1.27 | .209 |
| Distractor Type: low-calorie × Group: HC | 0.93 | 0.35 | 132.00 | 2.69 | .008 |
| Distractor Type: neutral × Group: HC | 0.88 | 0.35 | 132.00 | 2.56 | .012 |

Note. HC = healthy controls. Age was included as a covariate.

# **Supplementary Table S10**

*Descriptive statistics of early posterior negativity of neutral targets.*

| Distractor Type | AN  *M* (*SD*) | HC  *M* (*SD*) |
| --- | --- | --- |
| High-calorie | 0.62 (2.63) | -0.54 (1.93) |
| Low-calorie | 1.14 (2.10) | -0.19 (2.09) |
| Neutral | -0.06 (2.39) | -0.87 (2.21) |

*Note.* Mean amplitudes and standard deviations of early posterior negativity mean voltages (μV) in the time window of 220 ms to 340 ms after stimulus onset for correctly identified neutral targets according to *Group* and *Distractor Type*. AN = Anorexia nervosa, *n* = 32, HC = healthy controls, *n* = 28.

# **Supplementary Table S11**

*Results of the linear mixed model testing the main effects of group and distractor type on early posterior negativity amplitudes of neutral targets.*

|  | Estimate | *SE* | *df* | *t* | *p* |
| --- | --- | --- | --- | --- | --- |
| Intercept | -3.63 | 2.32 | 60.29 | -1.56 | .123 |
| Distractor Type: low-calorie | 0.44 | 0.20 | 120.00 | 2.22 | .028 |
| Distractor Type: neutral | -0.52 | 0.20 | 120.00 | -2.64 | .010 |
| Group: HC | -1.26 | 0.52 | 60.00 | -2.43 | .018 |
| Age | 0.28 | 0.15 | 60.00 | 1.84 | .070 |

Note. HC = healthy controls. Age was included as a covariate.

# **Supplementary Table S12**

*Descriptive statistics of P300 of neutral targets.*

| Distractor Type | AN  *M* (*SD*) | HC  *M* (*SD*) |
| --- | --- | --- |
| High-calorie | 3.19 (3.31) | 2.78 (2.34) |
| Low-calorie | 4.04 (2.20) | 3.13 (2.88) |
| Neutral | 3.14 (3.06) | 2.78 (3.01) |

*Note.* Mean amplitudes and standard deviations of P300 peaks (μV) in the time window of 320 ms – 480 ms after stimulus onset for correctly identified neutral targets according to *Group* and *Distractor Type*. AN = Anorexia nervosa, *n* = 32, HC = healthy controls, *n* = 28.

# **Supplementary Table S13**

*Results of the linear mixed model testing the influence of distractor type on P300 amplitudes of neutral targets.*

|  | Estimate | *SE* | *df* | *t* | *p* |
| --- | --- | --- | --- | --- | --- |
| Intercept | 0.77 | 3.02 | 60.26 | 0.26 | .798 |
| Distractor Type: low-calorie | 0.62 | 0.24 | 120.00 | 2.55 | .012 |
| Distractor Type: neutral | -0.02 | 0.24 | 120.00 | -0.08 | .937 |
| Age | 0.15 | 0.20 | 60.00 | 0.74 | .462 |

Note. Age was included as a covariate.

# **Supplementary Table S14**

*Descriptive statistics of early posterior negativity of targets after neutral distractors.*

| Target Type | AN  *M* (*SD*) | HC  *M* (*SD*) |
| --- | --- | --- |
| High-calorie | -1.38 (2.76) | -1.65 (2.40) |
| Low-calorie | -1.72 (2.52) | -2.09 (2.56) |
| Neutral | -0.06 (2.39) | -0.87 (2.21) |

*Note.* Mean amplitudes and standard deviations of early posterior negativity mean voltages (μV) in the time window of 220 ms to 340 ms after stimulus onset for correctly identified targets after neutral distractors according to *Group* and *Target Type*. AN = Anorexia nervosa, *n* = 32, HC = healthy controls, *n* = 28.

# **Supplementary Table S15**

*Results of the linear mixed model testing the main effects of group and target type on early posterior negativity amplitudes of targets.*

|  | Estimate | *SE* | *df* | *t* | *p* |
| --- | --- | --- | --- | --- | --- |
| Intercept | -6.34 | 2.66 | 60.18 | -2.39 | .020 |
| Target Type: low-calorie | -0.38 | 0.18 | 120.00 | -2.16 | .033 |
| Target Type: neutral | 1.07 | 0.18 | 120.00 | 6.01 | < .001 |
| Age | 0.32 | 0.17 | 60.00 | 1.83 | .072 |

Note. Age was included as a covariate.

# **Supplementary Table S16**

*Descriptive statistics of P300 of targets after neutral distractors.*

| Target Type | AN  *M* (*SD*) | HC  *M* (*SD*) |
| --- | --- | --- |
| High-calorie | 2.83 (3.15) | 2.60 (2.86) |
| Low-calorie | 2.64 (2.96) | 2.32 (2.98) |
| Neutral | 3.14 (3.06) | 2.78 (3.01) |

*Note.* Mean amplitudes and standard deviations of P300 peaks (μV) in the time window of 320 ms – 480 ms after stimulus onset for correctly identified targets after neutral distractors according to *Group* and *Target Type*. AN = Anorexia nervosa, *n* = 32, HC = healthy controls, *n* = 28.

# **Supplementary Table S17**

*Visual analogue scale values of hunger, mood and arousal before and after the rapid serial visual presentation task.*

|  | AN (*n* = 33) | | HC (*n* = 32)^a^ | |
| --- | --- | --- | --- | --- |
|  | pre | post | pre | post |
| Hunger | 8.52 (13.31) | 13.48 (20.22) | 18.78 (21.37) | 30.81 (26.36) |
| Mood | 60.82 (15.72) | 50.55 (13.87) | 77.81 (14.73) | 59.47 (20.09) |
| Arousal | 37.36 (23.95) | 45.48 (25.66) | 21.81 (22.13) | 27.06 (26.12) |

Note. Values represent mean (*SD*). Visual analogue scale: 1-100. Hunger: “How hungry are you at the moment?”; “not at all” and “very much”; mood: “How do you feel at the moment?”, “very bad” and “very good”, and arousal “How tense are you at the moment?” “not at all” and “very much”. AN = Anorexia nervosa, HC = healthy controls.

^a^Due to technical issues data of one participant in the HC group is missing.

# **Supplementary Table S18**

*Results of the linear mixed model testing the influence of time and group on hunger.*

|  | Estimate | *SE* | *df* | *t* | *p* |
| --- | --- | --- | --- | --- | --- |
| Intercept | 15.22 | 3.42 | 79.06 | 4.46 | < .001 |
| Time: pre | -8.45 | 2.15 | 65.00 | -3.94 | < .001 |
| Group: HC | 13.80 | 4.62 | 65.00 | 2.98 | .004 |

Note. HC = healthy controls.

# **Supplementary Table S19**

*Results of the linear mixed model testing the influence of time and group on mood.*

|  | Estimate | *SE* | *df* | *t* | *p* |
| --- | --- | --- | --- | --- | --- |
| Intercept | 50.55 | 2.79 | 102.15 | 18.14 | < .001 |
| Time: pre | 10.27 | 2.72 | 65.00 | 3.77 | < .001 |
| Group: HC | 8.92 | 3.97 | 102.15 | 2.25 | .027 |
| Time: pre × Group: HC | 8.07 | 3.88 | 65.00 | 2.08 | .042 |

Note. HC = healthy controls.

# **Supplementary Table S20**

*Results of the linear mixed model testing the influence of time and group on arousal.*

|  | Estimate | *SE* | *df* | *t* | *p* |
| --- | --- | --- | --- | --- | --- |
| Intercept | 44.78 | 4.01 | 79.79 | 11.17 | < .001 |
| Time: pre | -6.71 | 2.58 | 65.00 | -2.60 | .011 |
| Group: HC | -16.99 | 5.41 | 65.00 | -3.14 | .003 |

Note. HC = healthy controls.

# **References**

1. Hilbert A. Eating Disorder Examination für Kinder: Deutschsprachige Übersetzung. Tübingen: dgvt-Verlag; 2016.

2. Van Strien T, Oosterveld P. The children’s DEBQ for assessment of restrained, emotional, and external eating in 7- to 12-year-old children. Int J Eat Disord. 2008;41:72–81. https://doi.org/10.1002/eat.20424
